# Whole-genome/exome analysis of circulating tumor DNA and comparison to tumor genomics from patients with heavily pre-treated ovarian cancer: subset analysis of the PERMED-01 trial

**DOI:** 10.3389/fonc.2022.946257

**Published:** 2022-07-29

**Authors:** Renaud Sabatier, Séverine Garnier, Arnaud Guille, Nadine Carbuccia, Jihane Pakradouni, José Adelaide, Magali Provansal, Maria Cappiello, Frédérique Rousseau, Max Chaffanet, Daniel Birnbaum, Emilie Mamessier, Anthony Gonçalves, François Bertucci

**Affiliations:** ^1^ Aix-Marseille Univ, Inserm, CNRS, Institut Paoli-Calmettes, CRCM—Predictive Oncology Laboratory, Marseille, France; ^2^ Aix-Marseille Univ, Inserm, CNRS, Institut Paoli-Calmettes—Department of Medical Oncology, CRCM, Marseille, France; ^3^ Department of Clinical Research and Innovation, Institut Paoli-Calmettes, Marseille, France

**Keywords:** ovarian cancer, circulating tumor DNA, low-coverage whole-genome sequencing, copy number alterations, whole-exome sequencing, mutations, tumor mutation burden

## Abstract

**Introduction:**

The poor prognosis of ovarian carcinoma (OvC) is due to the advanced stage at diagnosis, a high risk of relapse after first-line therapies, and the lack of efficient treatments in the recurrence setting. Circulating tumor DNA (ctDNA) analysis is a promising tool to assess treatment-resistant OvC and may avoid iterative tissue biopsies. We aimed to evaluate the genomic profile of recurrent heavily pre-treated OvC.

**Methods:**

We performed tumor panel-based sequencing as well as low-coverage whole-genome sequencing (LC-WGS) of tumor and plasma collected in patients with ovarian cancer included in the PERMED-01 trial. Whole-exome sequencing (WES) data of plasma samples were also analyzed and compared to mutation and copy number alteration (CNA) tumor profiles. The prognostic value [progression-free survival (PFS)] of these alterations was assessed in an exploratory analysis.

**Results:**

Tumor and plasma genomic analyses were done for 24 patients with heavily pretreated OvC [67% high-grade serous carcinoma (HGSC)]. Tumor mutation burden was low (median 2.04 mutations/Mb) and the most frequent mutated gene was *TP53* (94% of HGSC). Tumor CNAs were frequent with a median of 50% of genome altered fraction. Plasma LC-WGS and WES detected ctDNA in 21/24 cases (88%) with a median tumor fraction of 12.7%. We observed a low correlation between plasma and tumor CNA profiles. However, this correlation was significant in cases with the highest circulating tumor fraction. Plasma genome altered fraction and plasma mutation burden (*p* = 0.011 and *p* = 0.041, respectively, log-rank tests) were associated with PFS.

**Conclusions:**

Combination of LC-WGS and WES can detect ctDNA in most pre-treated OvCs. Some ctDNA characteristics, such as genome altered fraction and plasma mutation burden, showed prognostic value. ctDNA assessment with LC-WGS may be a promising and non-expansive tool to evaluate disease evolution in this disease with high genomic instability.

**Clinical Trial Registration:**

https://clinicaltrials.gov/ct2/show/NCT02342158, identifier NCT02342158.

## Introduction

Ovarian carcinomas (OvCs) are the fifth leading cause of death by cancer in women (1). This poor prognosis (5-year survival close to 30%) is due to the advanced stage at diagnosis, a high recurrence rate, and low treatment efficacy in the platinum-resistant setting (median overall of 1 year) (2, 3). Great efforts are thus warranted to improve our knowledge of platinum-resistant tumors and identify new therapeutic targets for this population.

Platinum-resistant OvCs are heterogeneous, and their evolution is poorly understood by usual clinicopathological criteria (3). This heterogeneity of morphologically similar tumors suggests that biology may be heterogeneous not only between patients but also between disease sites (4). Previous efforts performed to study these tumors have not led to outcome improvement, maybe because they were focused on limited tumor samples not reflecting the biological complexity of the disease (5, 6). Analysis of circulating tumor materials may improve assessment of disease biological heterogeneity (7, 8). Moreover, tumor sampling using surgical procedures or guided biopsies can be too invasive for heavily pre-treated patients, and blood sampling should be more acceptable from an ethical point of view.

Mutation and copy number alteration (CNA) profiles of newly diagnosed OvC have been widely described with high genomic instability as a hallmark of OvC (9). Low-coverage whole genome sequencing (LC-WGS) can efficiently identify tumor alterations in OvC (10) but data are scarce concerning platinum-resistant tumors. Whole-exome sequencing (WES) of ovarian cancer tissues has already been reported (9, 11), but no data with this technology are available for plasma analysis. Plasma WES may be of interest to complete LC-WGS profiles.

By combining LC-WGS and WES of plasma samples from heavily pre-treated patients with OvC enrolled in a prospective cohort, we aimed to develop a non-invasive comprehensive genomic profile of these platinum-resistant tumors.

## Materials and methods

### Patients’ selection and study design

All patients analyzed in this work were included in the PERMED-01 study (NCT02342158). PERMED-01 was a prospective non-controlled trial aiming to evaluate the number of patients with advanced cancer for whom identification of actionable genetic alterations in tumor samples could lead to the delivery of matched therapies. Among others, secondary objectives included description of molecular alterations and their correlations to clinicopathological features and survival, as well as analysis of liquid biopsies such as circulating tumor DNA (ctDNA) analysis. Various tumor types were enrolled including breast cancer, lung, gastro-intestinal, prostate, and gynecological cancers. Details of inclusion criteria, patients’ selection, and clinical results can be found elsewhere (12). This subset study was focused on patients with OvC and with tumor, plasma, and germline samples available.

### Tumor and germline sample collection and DNA extraction

We collected germline and genomic DNA for all patients included in this study. All genomic analyses were performed on *de novo* tumor biopsies or resections. Only frozen samples with at least 20% of tumor cells as assessed by the pathologist were retained for analysis. Tumor DNA and germline DNA were extracted as previously described (12). DNA concentration was quantified with the Qubit fluorometer (Qubit dsDNA-BR kit, ThermoFisher Scientific™, Waltham, MA, USA) according to the manufacturer’s instructions. DNA was then stored at −20°C before further analyses.

### Plasma sample collection and cell-free DNA extraction

Peripheral blood was collected in four 5-ml EDTA tubes at inclusion. Blood was centrifuged twice within 2 h after venipuncture and plasma was stored at −80°C. Cell-free DNA was isolated from plasma using the Maxwell^®^ (Promega™, Madison, WI, USA) and Maxwell^®^ RSC circulating cell-free DNA Plasma Kit (Promega™) and quantified by the Qubit fluorometer (QuBit HS dsDNA kit, ThermoFisher Scientific™), according to the manufacturer’s instructions. Cell-free DNA was then stored at −20°C before further analyses.

### Low-coverage whole-genome sequencing

We explored copy number abnormalities, using low-coverage whole-genome sequencing analysis (LC-WGS). Libraries were constructed using the commercially available MicroPlex Library Preparation Kit v2 (Diagenode™, Liège, Belgium) in accordance with the manufacturer’s instructions. The cfDNA input was 5 ng per library preparation. The quality and quantity of each library were evaluated by the Agilent 2200 TapeStation System (Agilent HS D1000 Assay Kit, Agilent™, Santa Clara, CA, USA) and the Qubit Fluorometer (QubitTM dsDNA BR Assay Kit, ThermoFisher Scientific ™), respectively. Each library was then reduced to 4 nM before being pooled in equimolar amounts (12 libraries per mix). All library mixes were sequenced on a NextSeq500^®^ Next-Generation Sequencer from Illumina (San Diego, CA, USA) with an average depth of coverage of 0.4×, generating readings of 2 * 75 base pairs (bp). We then determined the DNA fraction from tumor cells [tumor fraction (TF)] for each sample. Reads were aligned with the human reference genome (hg19) using the bwa software (version 0.7.15-r1140). Alignment was then processed to remove the duplicate sequences with the Picard software (version 2.9.2). A wig file containing the number of reads for regular intervals of 50,000 bp for tumor DNA and 500,00 bp for cfDNA was generated with the readCounter software. Finally, TF evaluation was obtained using the ichorCNA software (version 0.3.2). For plasma samples, *in silico* size selection was performed to limit cell-free DNA analysis to DNA fragments from 90 to 180 bp (13). We chose 500,000 bp to define intervals used by the readCounter software to limit copy number profile fragmentation.

For each sample, the genomic profile was established. To identify recurrent copy number alterations, we used the Genomic Identification of Significant Targets in Cancer (GISTIC) 2.0 algorithm (14), calculated by multiple random iterations, with an amplification/deletion threshold >0.9, confidence level 0.90, and a corrected threshold probability *q* < 0.25. We computed the percentage of concordance between plasma and tumor for each significant region. Gained regions were consistent if gained in both samples (copy number ≥3), and lost regions were consistent if lost in both samples (copy number ≤1). We identified driver genes in these CNAs by using Cancer Genome interpreter (CGI, https://www.cancergenomeinterpreter.org/home). Genome altered fraction (GAF) was defined as the sum of altered regions divided by the total number of regions after removing sexual chromosomes.

### Single-nucleotide mutation analyses

Tumor and germline DNA were sequenced using four chronologically extended home-made panels of genes as previously described (12). Sequence data were aligned to the human genome (UCSC hg19) and alignment processed as previously described (15). Genomic signature exposure was explored according to Macintyre’s algorithm (10).

Cell-free DNA WES was performed using the Agilent SureSelectXT V6 target enrichment system. Paired-end sequencing was performed using the NextSeq 500 (2 × 150 cycles; Illumina). All bioinformatics analyses related to WES were performed as previously described (16). Reads were aligned with the human reference genome (hg19) using the bwa software (version 0.7.15-r1140). Alignment was then processed to remove the duplicate sequences with the Picard software (version 2.9.2). Somatic variant calling was done with Mutect2, and annotation was performed with Annovar.

Plasma WES and LC-WGS data are available in **Supplementary Tables 5** and **6**.

### Statistical analyses

Patient’s characteristics were summarized by frequency counts and percentages for categorical variables and medians and ranges for continuous variables. Progression-free survival (PFS) was defined as the delay from tumor biopsy to disease progression or death, whatever occurred first. Progression was defined according to Gynecological Cancer Intergroup (GCIC) criteria (17). Patients lost to follow-up or without any event were censored at the date of last contact. Correlation of PFS to plasma TF, plasma tumor mutation burden (TMB), and circulating GAF was performed using Cox proportional hazards regression. Hazard ratios (HRs) with their 95% confidence interval (95% CI) were provided and the null assumption (HR = 1) was assessed using the Wald’s test. For binary variables, comparisons between groups were estimated using the Kaplan–Meier method and subgroups were compared by log-rank test. In order to search CNAs associated with survival, we performed a supervised analysis in patients diagnosed with high-grade serous carcinoma (HGSC). We used the median PFS as threshold for dichotomization (above and below the median). For each region, we compared the frequency of gain or loss between the two groups (short vs. long PFS) with a Fisher’s exact test. The statistical analyses were carried out using SAS version 9.4 (SAS Institute, Cary, NC, USA) with a nominal level of statistical significance (two-tailed) set to 0.05 and using R (version 3.5.1; http://www.cran.r-project.org/). This study was conducted in compliance with the Reporting recommendations for tumor marker prognostic studies (REMARK) criteria (18) (see **Supplementary checklist**).

## Results

### Ovarian cancer cohort characteristics

**Figure 1:**
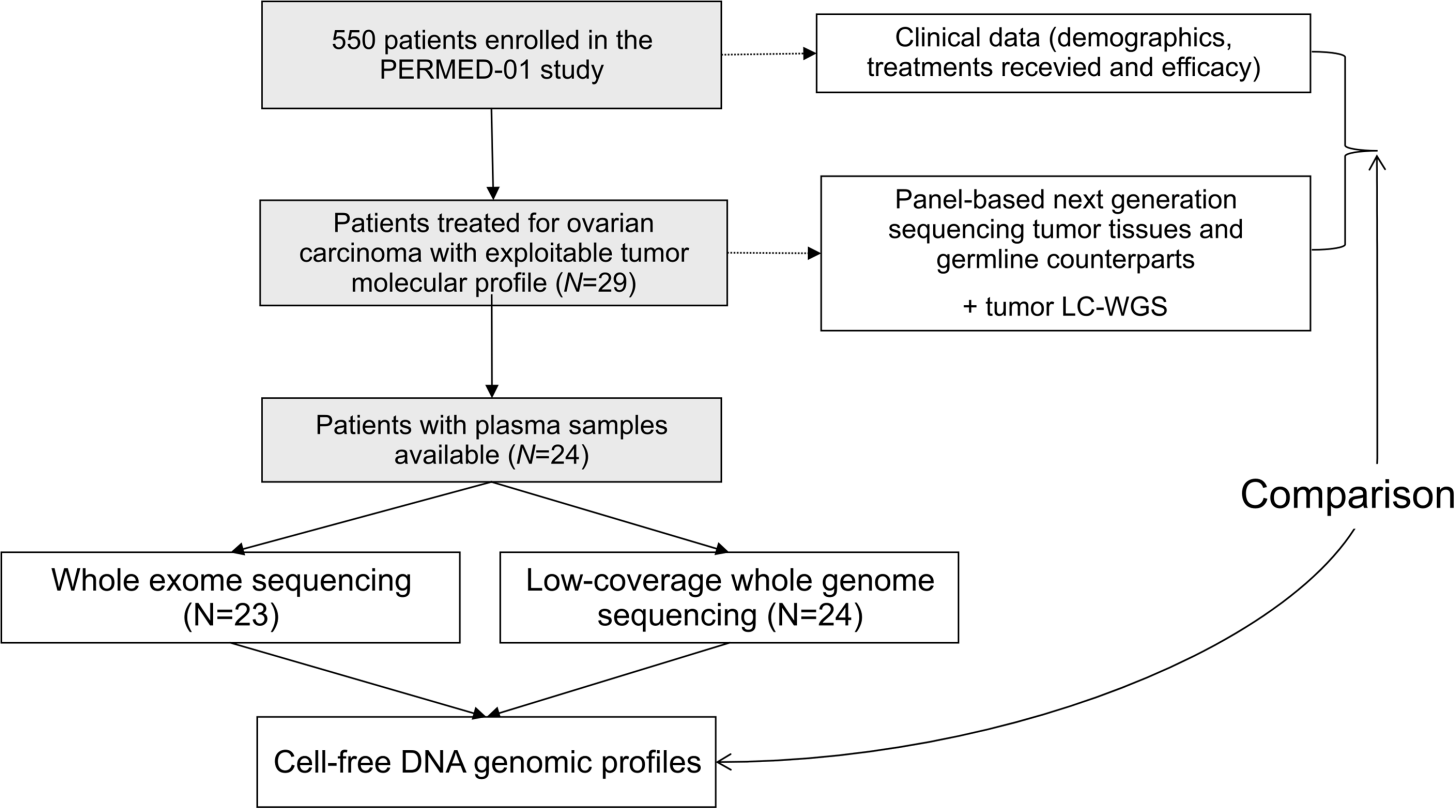
Study flow diagram.

From November 2014 to September 2019, 550 patients were enrolled in the PERMED-01 trial, including 32 patients with OvC. Tumor and germline molecular profiles were available for 29 OvC cases. Of them 24 had plasma available (**Figure 1**). Clinicopathological features of these 24 patients are detailed in **Table 1**. Median age of patients at inclusion was 54 years old (range, 21–71). Most of patients had HGSC (67%). Patients had received a median of three prior lines of chemotherapy and 92% were platinum-resistant at time of inclusion, i.e., experienced disease progression less than 6 months after completion of the last platinum-based chemotherapy line.

**Table 1:** Clinical and molecular characteristics of patients included in the ovarian cancer cohort of the PERMED-01 trial.

Number of patients	24
**Median age (years, range)**	54.3 [21.7–71.1]
**Histology**	
- HGSC - LGSC - Clear cell - Endometrioid - Carcinosarcoma	16 3 3 1 1
**Site of baseline biopsy**	
- Peritoneum - Lymph nodes - Liver - other	9 (38%) 7 (29%) 6 (25%) 2 (8%)
**Platinum-resistant at inclusion**	22 (92%)
**Priori lines of chemotherapy**	
- Median (range)	3 (1-6)
**Most frequent somatic alterations**	
- *TP53* mutation - *ARID1A* mutation - *ARID1A* loss - *PI3KCA* mutation	15 (63%), Including 15/16 HGSC (92%) 2 (8%, all clear cell) 2 (8%, all LGSC) 2 (8%, 1 clear cell, 1 endometrioid)
**Actionable genetic alterations**	10 (42%)
***Genomic-guided therapies***	3 (13%)
- Lapatinib - Sorafenib - Everolimus	*ERBB2* amp, PD *KRAS* mut, SD *PIK3CA* mut, PR

Data are expressed as N (%) unless otherwise specified. HGSC, high-grade serous cancer; LGSC, low-grade serous cancer; PD, progressive disease; SD, stable disease; PR, partial response.

Similar to what has been shown in the entire PERMED-01 study (12), actionable genomic alterations were identified in 10 (42%) patients and three patients received genomic-guided therapies. Median PFS was 4.83 months (range 0.66–25.26) (**Supplementary Figure 1**).

### Tumor genomic profiles

Panel-based sequencing of the 24 tumor tissues [median depth 751× (range, 463–3,371)] identified a median of four mutations per sample (1–8) and all cases displayed a low tumor mutational burden [TMB; median 2.04 mut/Mb (range, 0.51–4.66)]. Most frequent somatic mutations involved *TP53* (*N* = 15, all HGSC), followed by *ARID1A* (*N* = 2, all clear cell cancer), and *PIK3CA* (*N* = 2, one clear cell and one endometrioid carcinoma). Five patients harbored mutations in genes involved in homologous recombination (**Supplementary Table 1**).

**Figure 2:**
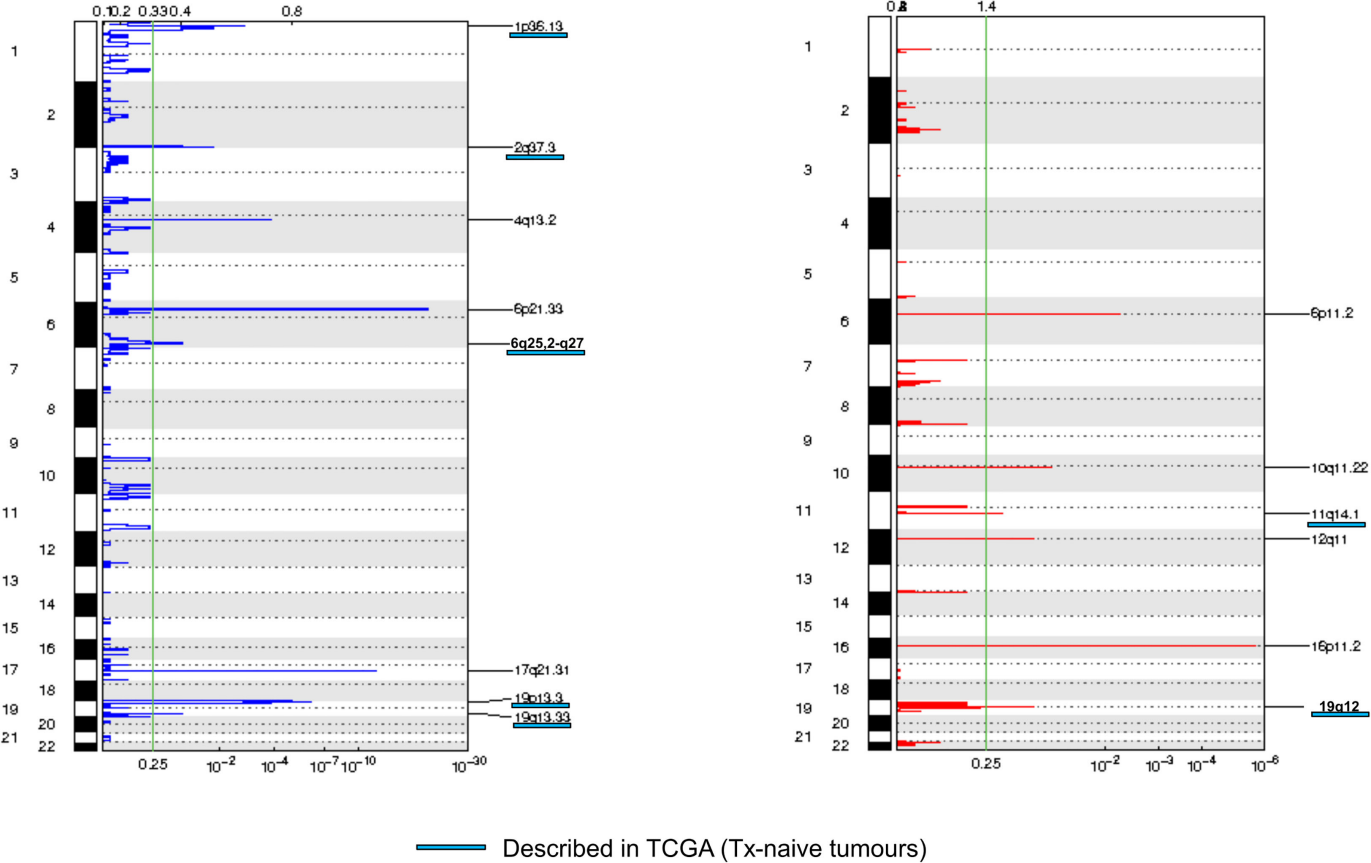
Frequency plot of recurrent copy number alterations identified in tumor samples using the GISTIC algorithm. Frequencies of losses (left) and gains (right) are plotted as a result of chromosome location. *x*-axis: top = log-scale ratio; bottom = *q*-values. Alterations described in TCGA are underlined in blue. Green lines represent the threshold for significance.

We then assessed tumor CNA profiles by using LC-WGS on tumor DNA (median depth 0.6×, range, 0.49–0.84). Median tumor fraction was 32.45% (range, 4.53–72.98). GAF was variable across cases (median 50%, range 12%–88%). GISTIC analysis identified several recurrent altered regions (**Figure 2**), including eight regions with loss/deletions and six regions with gains/amplifications (**Supplementary Table 2**). Among them, seven were already described in chemotherapy-naive tumors from TCGA (9).

**Figure 3:**
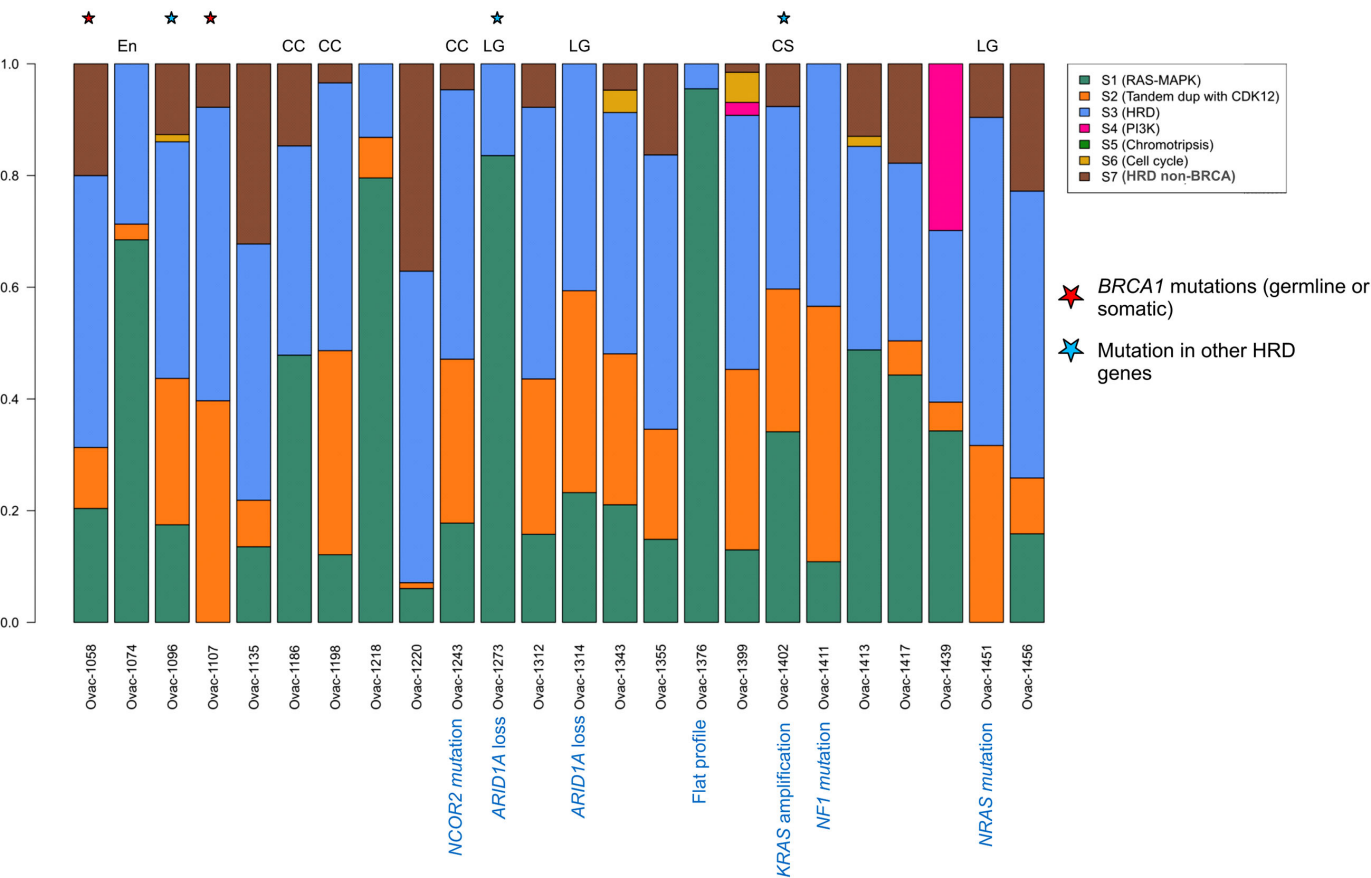
Copy number signature exposures for each patient. Stacked bar plots representing copy number signature exposures according to (10). Patients were gathered according to tumor pathological subtypes. CC, clear cell; LG, low-grade serous; CS, carcinosarcoma; En, endometrioid.

Using a previously published algorithm (10), we then explored the CNA signature profiles of each tumor (**Figure 3**). As expected, most HGSCs displayed HRD (homologous recombination deficiency) profiles. The three HGSC cases with somatic or germline mutations in HRD-related genes had signatures S3 (BRCA-related HRD) as dominant signature. Of note, the only case with *BRCA* mutation with a non-HRD dominant signature was a carcinosarcoma with *KRAS* amplification and a RAS-MAPK dominant signature. Signature S1 (RAS-MAPK pathway alteration) was dominant in five HGSC cases including one with *FGFR4* mutation and *FGFR1* gain, and a case with a flat profile probably related to its low cellularity (20%). Signature S2 (Tandem duplication) was dominant in the last HGSC also harboring *NF1* mutation. Profiles were more heterogeneous in other subtypes. Two of three clear cell tumors and two of three low-grade serous cases had an HRD-related dominant signature. Signature S1 was dominant in one clear cell OvC, one LGSC, and the endometrioid tumor.

### Plasma genomic alterations

We first assessed the fraction of cell-free DNA from ovarian cancer in each of the 24 cases. LC-WGS of cell-free DNA (median depth 1.07×, range 0.53–1.41) detected ctDNA in 21 of 24 cases (87.5%). Median tumor fraction was 12.65% (range 5.50%–41.41%). Plasma tumor fraction was correlated with tissue tumor fraction (Pearson correlation coefficient = 0.67, *p* < 0.001, **Figure S2A**). Median GAF was 15.2% (range, 5.8–81.9).

**Figure 4:**
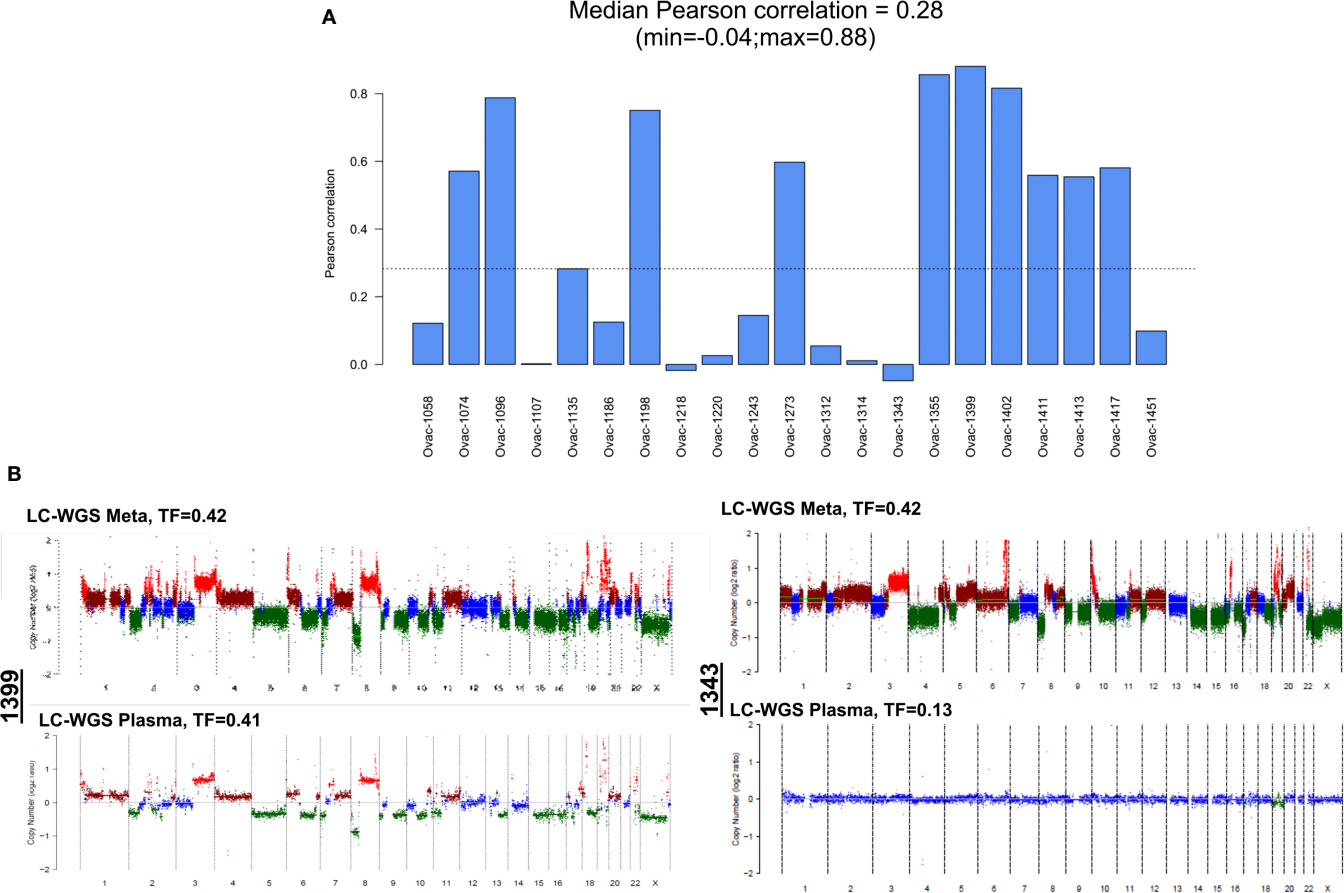
Correlation between plasma and tumor copy number alteration profiles determined by LC-WGS in tumor with baseline plasma tumor fraction (TF) >0. **(A)** Pearson correlation for each patient. **(B)** Examples of tumor and plasma CNA profiles in two cases (1399 and 1343).

Comparison of CNA profiles in plasma samples (in cases with TF > 0) and tumor counterparts showed a low correlation (**Figure 4A**). However, some cases had very similar CNA profiles with almost all tumor CNAs also detected in plasma (**Figure 4B**). Correlation between plasma and tumor CNA profiles tended to be higher in cases with the highest plasma TF (Pearson correlation coefficient 0.55, *p* = 0.0095, **Figure S2B**).

**Figure 5:**
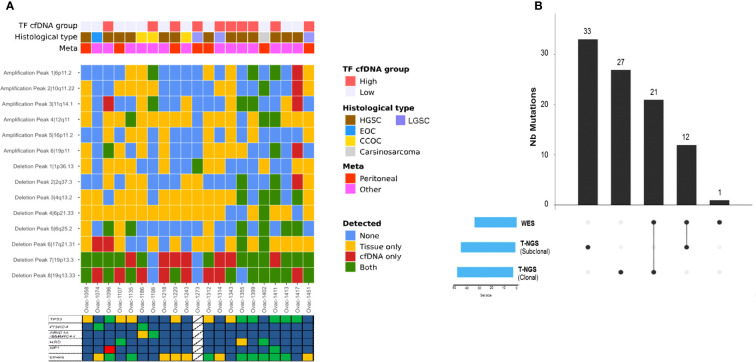
Copy number alterations and mutation profiles of ctDNA. **(A)** Heatmap of genomic alterations identified in tumors and plasma counterparts. TF, tumor fraction; HGSC, high-grade serous carcinoma; LGSC, low-grade serous carcinoma; EOC, endometrioid ovarian cancer; CC, clear cell. Stars represent cases with at least one tumor alteration identified in plasma. **(B)** Upset chart showing the comparison of single-nucleotide variants identified using WES versus t-NGS.

All but one (93%, five of six amplifications and all deletions) recurrent altered region identified with tumor GISTIC analysis were also observed in at least one plasma sample (**Supplementary Table 2**). Deletions in chromosome 19 (19p13.3 and 19q13.33) were observed in most plasma samples (**Figure 5A**). Eight of 18 patients with plasma 19q13.33 deletion also displayed this deletion in tumor counterparts. A similar observation (7 of 19) was made for 19p13.3 deletion. Correlations between tumor and plasma were independent from baseline plasma TF, pathological subtype, and site of tumor biopsy (**Figure 5A**).

### Whole-exome analysis of plasma samples

We then explored plasma samples and germline counterparts with WES (median depth 123.71×, range 80.68–242.55). We encountered technical issues and were not able to perform WES in one case. TF defined by WES was lower than LC-WGS TF (median 0.1, range 0.1–0.61) and was correlated to LC-WGS TF (**Supplementary Figure 3A**). Plasma CNA profiles defined by LC-WGS were closer to tumor than plasma WES profiles (**Supplementary Figure 3B**).

We observed a median of 21 somatic mutations (range, 5–115) with all cases displaying a low mutational load (median 0.13 mut/Mb, range 0–0.99). Plasma TMB was not correlated to tumor TMB assessed by panel-based sequencing (Pearson correlation 0.13, **Supplementary Figure 3C**). Among all mutations detected, some involved the 60 genes also evaluated in tumors with our panel-based sequencing (**Figure 5B**). Thirty-three tumor mutations were detected in plasma (including 21 clonal mutations), whereas 60 (33 clonal) were not identified by WES. One *NF1* somatic mutation not detected by tumor sequencing was identified with plasma WES. When combining plasma LC-WGS and WES, we observed at least one tumor alteration in 21 of 24 patients (88%) including the three cases with LC-WGS TF equal to 0, who had circulating somatic mutation detected by WES. Circulating variant allele fraction was 3% or lower in the three cases, under the usually admitted detection threshold of circulating CNAs by LC-WGS. Actionable genetic alterations were identified in plasma in all three patients who received genomic-guided therapies.

Moreover, WES analysis allowed the identification of specific molecular processes associated with genomic instability. We identified a median of 5.5 large-scale state transitions (range 1–14) and a median of five loss-of-heterozygosity events (range, 0–14). The incidence of these events was similar between high-grade serous tumors and other pathological subtypes (*p* = 0.85 and 0.76, respectively).

It is of note that germline WES allowed identification of one additional *TP53* mutation with a variant allele fraction in germline DNA equal to 7.7%, and a similar frequency (8.2%) in tumor DNA. This mutation was related to clonal hematopoiesis in a patient who subsequently (21 months later) developed acute myeloid leukemia with complex karyotype. She died of leukemia 10 months after diagnosis.

### Correlation of plasma genomic alterations to clinical outcome

**Figure 6:**
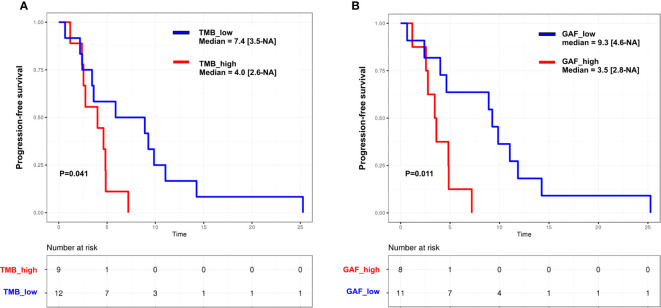
Kaplan–Meier curves for progression-free survival in the whole cohort according to **(A)** tumor mutation burden (TMB) and **(B)** genome altered fraction (GAF). Groups are split according to the median. *p*, log-rank test.

We then performed an exploratory assessment of the impact of ctDNA features on PFS. PFS was correlated with plasma TMB (HR = 8.6; 95% CI [1.4–52]) and circulating GAF (HR = 8.9; 95% CI [0.91–87]) (**Supplementary Table 3**). Median PFS was 4.0 (95% CI [2.6–NA]) months in patients with high (higher than the median) plasma TMB vs. 7.4 [3.5–NA] months in low TMB cases (*p* = 0.041), and 3.5 [2.8–NA] vs\. 9.3 [4.6–NA] months in cases with high and low GAF (*p* = 0.011), respectively (**Figure 6**). Similar trends were observed in the HGSC subset (**Supplementary Table 3**). Of note, neither pathological subtypes (HGSC vs. others, HR = 1.48, 95% CI [0.59–3.61]) nor number of prior lines of chemotherapy (<3 vs. ≥3; HR = 0.87, 95% CI [0.36–2.09]) were correlated to PFS in our set.

Finally, a supervised analysis of CNAs associated with shorter PFS was conducted in the HGSC subset to avoid subtype-specific bias. This exploratory analysis identified three genomic regions differentially altered between cases with short and long PFS (split on the median). Two regions were deleted in case with short survival: 6q11.1-6q27 (including *ESR1, FOXO3, ARID1B, UTRN, PRMD1*, and *PNRC1*) and 8p23.3-8p23.1. A large part of chromosome 8 [from 8p23.1 to 8q24.3 (including *FGFR1* and *MYC*)] was observed as amplified in cases with short PFS (**Supplementary Table 4**).

## Discussion

We show that low-coverage WGS and WES are feasible in plasma from heavily pre-treated OvC. Even though plasma analyses cannot reflect a comprehensive genomic profile of tumor samples, some disease characteristics can be identified in 88% of patients and some ctDNA features are associated with prognosis.

We describe here a comprehensive genomic analysis of the OvC subset of the PERMED-01 study. PERMED-01 showed that actionable genomic alterations can be identified in more than half of patients with pre-treated metastatic carcinoma (12). However, as also described in other genomic-guided treatment trials (19–21), less than 20% of patients received therapies matched with their molecular alterations, and only 1 of 20 patients experienced clinical benefit with this therapy. Similar results were observed in the current OvC cohort with only three patients receiving matched therapies and one displaying partial response. Most frequent genomic alterations identified in this heavily pre-treated dataset were consistent to usually described alterations. High-grade serous carcinomas were *TP53*-mutated and displayed high genomic instability (9). *ARID1A* and *PIK3CA* alterations were frequent in other subtypes (22–26). Using LC-WGS, we observed that most of the tumors displayed an HRD profile, consistent with previous observations with the same technologies (10). Patterns of chromosomal instability and HRD can also be identified in plasma from patients with OvC and are able to differentiate malignant serous tumors from benign adnexal masses (27). This may also allow using HRD detection in plasma analysis instead of tumor-based assays in order to prescribe PARP inhibitors that are routinely recommended both in the first-line and in recurrent setting (28–31). In our analysis, plasma and tumor CNA profiles were highly correlated in cases with the highest tumor fraction. ctDNA genomic instability was similar to that was observed in the tumor counterpart. Moreover, even in cases with low TF, we were able to identify at least one tumor CNA in 85% of ctDNA samples, with 19p13.3 and 19q13.33 deletions as the more frequent shared alterations. The 19q13.33 amplicon comprises *BAX*. The corresponding protein is involved in p53-induced apoptosis and low BAX expression is correlated to platinum resistance by upregulation of the Bcl2/Bcl-xL axis (32–35). 19p13.3 deletion has been described in ovarian clear cell cancer (36). Within this locus, the most obvious candidate is *STK11*, a well-known tumor suppressor gene involved in Peutz-Jeghers syndrome. STK11 loss has already been shown in ovarian serous carcinoma and may favor mTOR activation (37).

Our observations concerning ctDNA detection by LC-WGS are in line with a recent analysis of 46 patients treated in the first-line setting (38). Main tumor CNAs were retrieved in plasma at diagnosis suggesting that plasma LC-WGS analysis can be used as a surrogate of tumor alterations. Our results also suggest that the higher the amount of genomic alterations [both at the mutation (TMB) and CNA (GAF) levels], the poorer the prognosis. This may lead to implement ctDNA features as prognostic tools that would help patients and clinicians to choose the most adapted therapeutic strategies. Even though the small size of the cohort precludes performing multivariate analysis, the fact that high-grade serous cases with the highest GAF or the highest TMB also tended to have the poorest PFS suggests that it was independent from the pathological classification.

Some genomic alterations were associated with PFS. Chromosome 6q contains several tumor suppressor genes, and its deletion has been described in various tumor types, including Luminal B breast cancer, one of the most aggressive breast cancer subtype (39). Chromosome 8q24 amplification, including *MYC*, is widely described to be associated with tumorigenesis, notably in serous ovarian cancer (9, 38). However, despite the fact that *MYC* mRNA expression was associated with survival in The Cancer Genome Atlas dataset (40), the correlation of *MYC* amplification to ovarian cancer prognosis remains unclear (41).

Another innovative aspect of our study concerns the WES assessments of plasma from patients with OvC. To our knowledge, we are the first to describe this technology in this setting. We identified at least one tumor mutation in 21/24 patients (88%), close to what we can expect with panel-based NGS in these tumors with low mutation burden (42), and to what has been described in pre-treated metastatic cancers (43). As already shown, plasma VAF was lower than tumor VAF, reflecting a lower tumor fraction in the bloodstream (44, 45). A disappointing result was the moderate capacity to identify clonal mutations (53% of *TP53* mutations). This can be explained by our choice to maximize the amount of data we could obtain with this technology by sequencing cell-free DNA samples with TF<10% as assessed with LC-WGS. This threshold of 10% was set to optimize mutation results in other diseases with higher mutation rates (46). A TF above 25% has also been described to improve ctDNA detection sensitivity and agreement with tumor WES in a meta-analysis of more than 300 patients with various cancer types (47). Nevertheless, it is worth noting that we identified circulating mutation in the three cases with TF equal to 0, with mutant allele fractions of 3% or lower, under the admitted sensitivity of LC-WGS. Moreover, we identified one somatic *NF1* mutation not observed in tumor tissue, suggesting polyclonal evolution in this patient. This polyclonal evolution has been widely described in other tumor types such as breast cancer (48).

Particular attention should be given to germline sequencing besides cell-free DNA WES. It not only increases specificity by filtering single-nucleotide polymorphisms, but also can detect preliminary signals of clonal hematopoiesis. Hence, one patient in our set harbored *TP53* mutation in germline DNA at the time of inclusion and developed acute myeloid leukemia less than 2 years later. Limiting analysis to tumor and cell-free DNA may have led to interpret this mutation as subclonal. Germline sequencing is thus of high interest, notably in patients with prior treatment with alkylating agents and/or anthracyclines at high risk of induced hematological myeloid malignancies (49, 50).

Our work has limitations. First, its small size limits the prognostic analyses. However, this sample size is close to what has been published in other tumor types (44, 45, 51). Second, to be more comprehensive, panel-based NGS on cell-free DNA would have been of interest for comparison to tumor NGS mutation profile. Third, single-tumor biopsies were performed in the PREMED-01 trial. Multisite biopsies would have allowed a deeper exploration of tumor spatial heterogeneity and correlation to plasma analysis. Fourth, PERMED-01 did not plan to collect subsequent plasma samples after matched therapies initiation. We thus were not able to analyze multiple time points and could not explore ctDNA kinetics after treatment initiation, which is known to be correlated to survival (52). We were also not able to analyze clonal evolution under therapeutic pressure (53). Moreover, the low tumor mutation burden in ovarian cancer did not allow exploring mutation signatures, such as BRCA-like and APOBEC signatures, by plasma WES as is can be done in other tumor types (46).

In conclusion, the combination of LC-WGS and WES can detect ctDNA in most pre-treated OvCs. Some ctDNA characteristics associated with a higher amount of genomic alterations, such as circulating GAF and plasma TMB, may be prognostic. ctDNA assessment with LC-WGS may be a promising and non-expansive tool to evaluate disease evolution in this disease with high genomic instability. Larger and prospective studies are required to confirm our observations.

## Data availability statement

The datasets presented in this study can be found in online repositories. The names of the repository/repositories and accession number(s) can be found in the article/**Supplementary Material**.

## Ethics statement

The studies involving human participants were reviewed and approved by Comité de Protection des Personnes Sud Méditerranée I and the French Agency Health authority (ANSM). Registration number NCT02342158. The patients/participants provided their written informed consent to participate in this study.

## Author contributions

RS, AnG, and FB designed the study; SG, NC, and JA performed ctDNA experiments; ArG performed all bioinformatics and statistical analyses; RS, MP, MCa, FB, and AnG enrolled patients and collected clinical data; JP was responsible of clinical trial management; all authors contributed to data interpretation, manuscript writing, and approval of the submitted version.

## Funding

This study was conducted under the sponsorship of Institut Paoli-Calmettes and was supported by SIRIC, label Ligue EL2016, and Centre d’Investigations Cliniques (CICp1409). The funding sources were not involved in the study design; in the collection, analysis and interpretation of data; in the writing of the report; and in the decision to submit the article for publication.

## Acknowledgments

We would like to thank the Institut Paoli-Calmettes Biobank (the IPC/CRCM/UMR 1068 Tumor Bank, that operates under authorization # AC-2007-33 granted by the French Ministry of Research (Ministère de la Recherche et de l’Enseignement Supérieur) for sample storage and extraction of tumor and germline DNAs.

## Conflict of interest

AnG declares nonfinancial support from Novartis (travel, accommodation, and meeting registration fees). RS declares research grants from EISAI and AstraZeneca; advisory board for Roche, GSK, and Novartis; non-financial support (travel, accommodation, and meeting registration fees) from Pfizer, Roche, GSK, BMS, and AstraZeneca.

The remaining authors declare that the research was conducted in the absence of any commercial or financial relationships that could be construed as a potential conflict of interest.

## Publisher’s note

All claims expressed in this article are solely those of the authors and do not necessarily represent those of their affiliated organizations, or those of the publisher, the editors and the reviewers. Any product that may be evaluated in this article, or claim that may be made by its manufacturer, is not guaranteed or endorsed by the publisher.

## Supplementary material

Supplementary Figure 1 Kaplan Meier curves for the whole population (n=23). **(A)** Progression-free survival (PFS). **(B)** Overall survival (OS).

Supplementary Figure 2 Plasma tumor fraction (assessed by LC-WGS) correlations with **(A)** tissue tumor fraction and **(B)** Pearson correlation coefficient between tumor and plasma copy number alterations.

Supplementary Figure 3 Comparison of genomic alterations identified by WES to LC-WGS and tumor-NGS data. **(A)** Correlation of plasma TF assessed by WES and plasma TF assessed by LC-WGS. **(B)** Correlation of copy number alterations (CNA) profiles identified by plasma LC-WGS and plasma WES (tumor CNA profile as reference). **(C)** Correlation of tumor mutation burden in tumor (NGS) and plasma (WES).

Supplementary Table 5 Plasma WES data.

Supplementary Table 6 Plasma low-coverage - WGS data.

## Abbreviations

OvC, ovarian carcinomas; CNA, copy number alterations; LC-WGS, low-coverage whole-genome sequencing; WES, whole-exome sequencing; ctDNA, circulating tumor DNA; GISTIC, Genomic Identification of Significant Targets in Cancer; PFS, progression-free survival; GCIC, Gynecological Cancer InterGroup; TF, tumor fraction; TMB, tumor mutation burden; HR, hazard ratio; 95% CI, 95% confidence interval; REMARK, Reporting recommendations for tumor marker prognostic studies; HGSC, high-grade serous carcinoma; LGSC, low-grade serous carcinoma; TCGA, the Cancer Genome Atlas; HRD, homologous recombination deficiency.
