# Supplementary figures and images for: Whole-genome/exome analysis of circulating tumor DNA and comparison to tumor genomics from patients with heavily pre-treated ovarian cancer: subset analysis of the PERMED-01 trial
Source: Front Oncol. 2022 Jul 29;12:946257. doi: 10.3389/fonc.2022.946257 (PMC9373051; doi:10.3389/fonc.2022.946257)

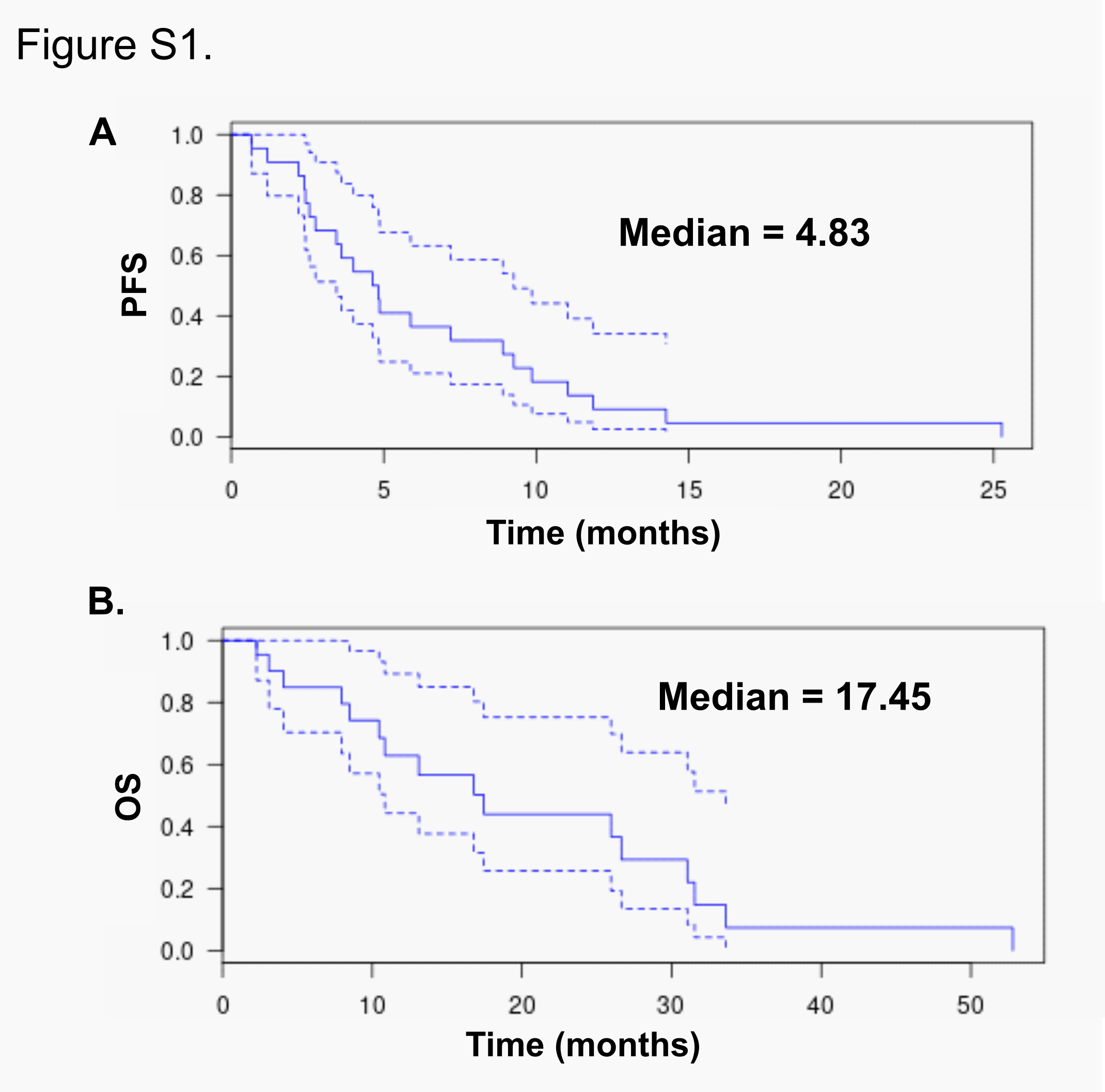

Supplement: Supplementary Figure 1 — Kaplan Meier curves for the whole population (n=23). (A) Progression-free survival (PFS). (B) Overall survival (OS). [file Image_1.tif]

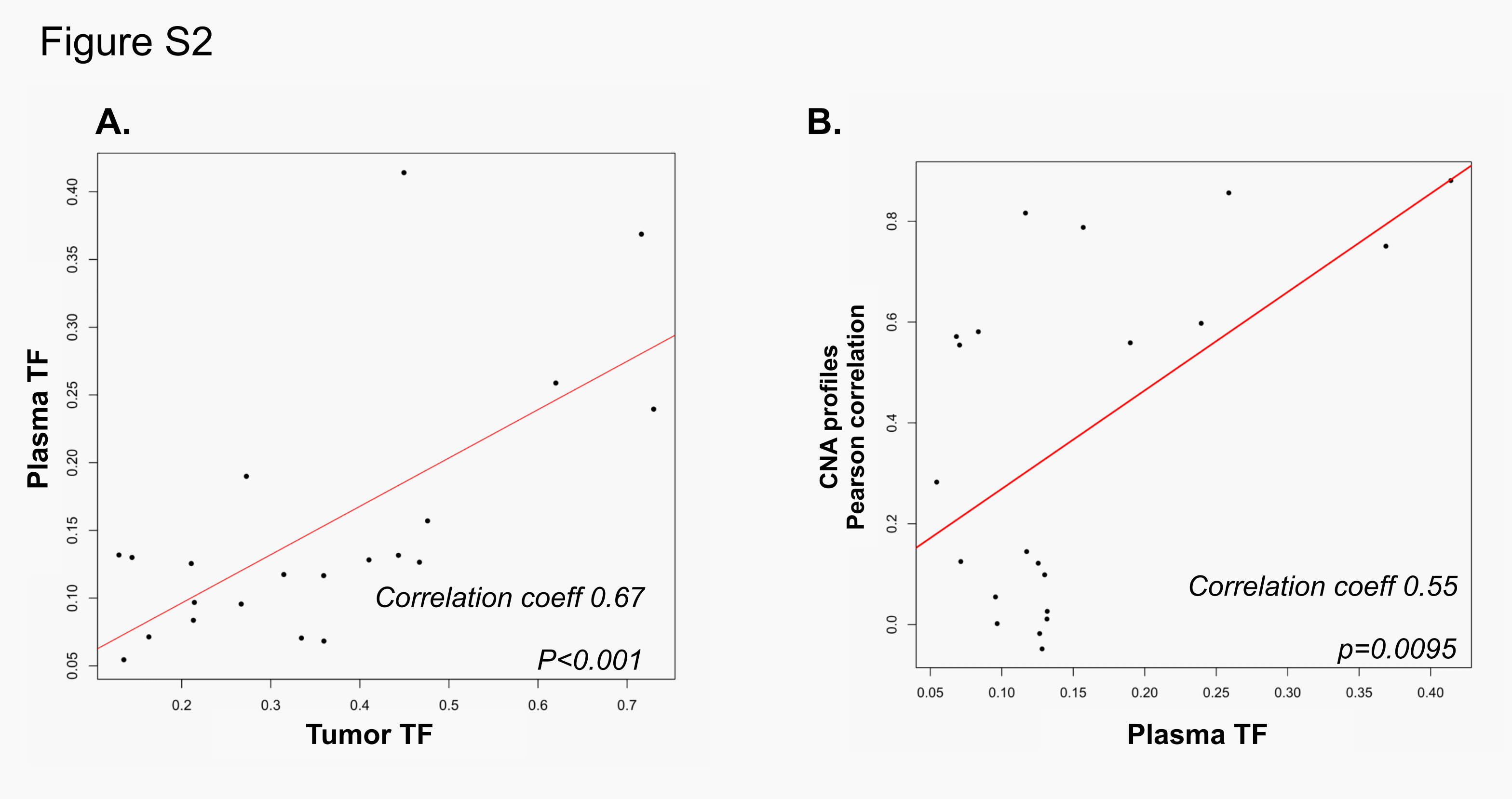

Supplement: Supplementary Figure 2 — Plasma tumor fraction (assessed by LC-WGS) correlations with (A) tissue tumor fraction and (B) Pearson correlation coefficient between tumor and plasma copy number alterations. [file Image_2.tif]

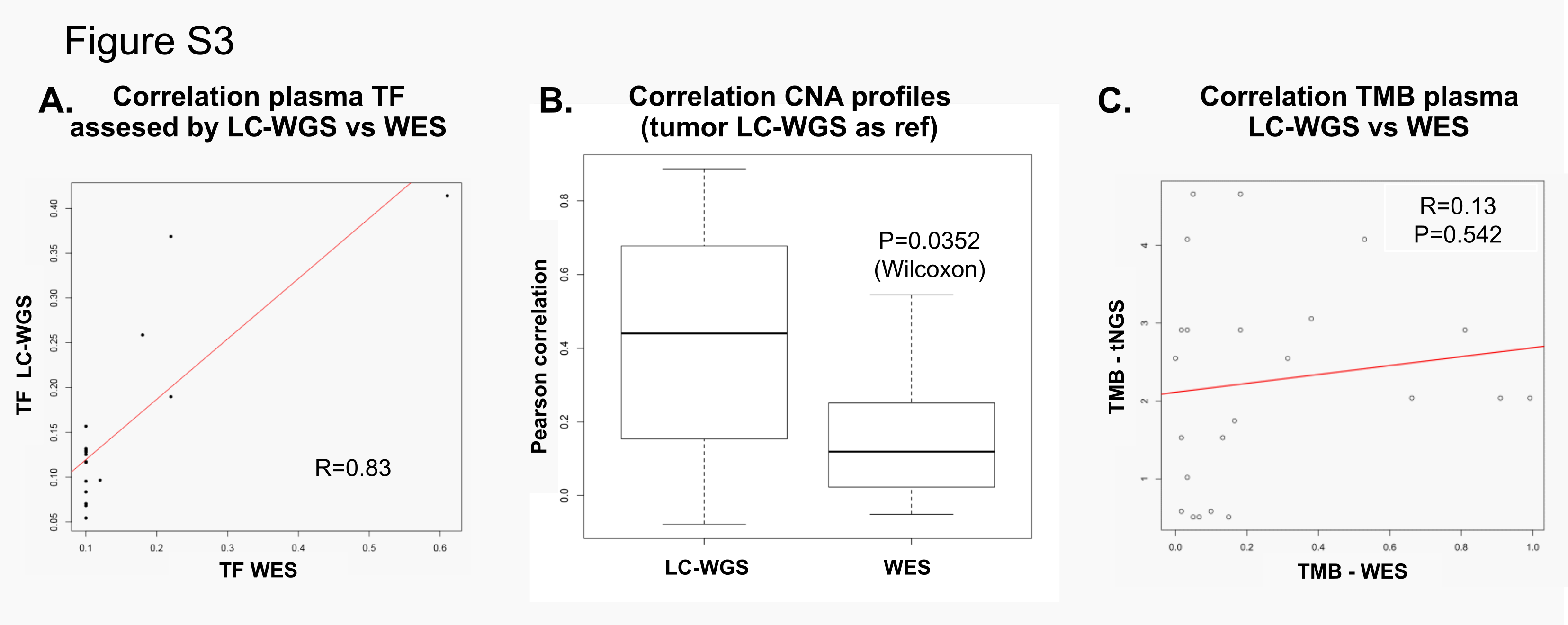

Supplement: Supplementary Figure 3 — Comparison of genomic alterations identified by WES to LC-WGS and tumor-NGS data. (A) Correlation of plasma TF assessed by WES and plasma TF assessed by LC-WGS. (B) Correlation of copy number alterations (CNA) profiles identified by plasma LC-WGS and plasma WES (tumor CNA profile as reference). (C) Correlation of tumor mutation burden in tumor (NGS) and plasma (WES). [file Image_3.tif]
